# Antibody validation for Western blot: By the user, for the user

**DOI:** 10.1074/jbc.RA119.010472

**Published:** 2019-12-09

**Authors:** Lakshmi Pillai-Kastoori, Sam Heaton, Steve D. Shiflett, Annabelle C. Roberts, Alejandra Solache, Amy R. Schutz-Geschwender

**Affiliations:** ‡ LI-COR Biosciences, Lincoln, Nebraska 68504; § Abcam Plc, Discovery Drive, Cambridge Biomedical Campus, Cambridge CB2 0AX, United Kingdom

**Keywords:** antibody, immunochemistry, antigen, western blot, protein domain, antibody validation, Immunoblot, irreproducibility crisis, Methodology, reproducibility

## Abstract

Well-characterized antibody reagents play a key role in the reproducibility of research findings, and inconsistent antibody performance leads to variability in Western blotting and other immunoassays. The current lack of clear, accepted standards for antibody validation and reporting of experimental details contributes to this problem. Because the performance of primary antibodies is strongly influenced by assay context, recommendations for validation and usage are unique to each type of immunoassay. Practical strategies are proposed for the validation of primary antibody specificity, selectivity, and reproducibility using Western blot analysis. The antibody should produce reproducible results within and between Western blotting experiments and the observed effect confirmed with a complementary or orthogonal method. Routine implementation of standardized antibody validation and reporting in immunoassays such as Western blotting may promote improved reproducibility across the global life sciences community.

## Introduction

Repeated observations are essential to the scientific method. They help to confirm that experimental observations are meaningful and reflect a biological truth when combined with robust statistical analysis. Despite this, however, the reproducibility of research findings has been a growing concern (1, 2). Sources of irreproducible research include incomplete reporting of experimental details, lack of reagent validation and controls, differences in analytic techniques, or measurements and differences in the interpretation of results (3, 4).

Many of the issues above arise from a common cause: researchers perform similar assays in many different ways. The lack of a unifying framework or set of standards is a clear barrier to reproducibility. In the life sciences, use of accepted standards can promote reproducibility by developing consensus-based methods that reduce unintentional differences between experiments, as well as improving data reporting practices to increase awareness of intentional differences between experiments (4–7).

Several peer-reviewed journal editors have commented on issues related to the lack of reproducible antibodies in life science research (8), and multiple initiatives are trying to look at ways in which antibodies can be standardized, including evaluation, protocols, and documentation. In 2016, the International Working Group for Antibody Validation (IWGAV)^3^ proposed guidelines for improving standards for antibody use and validation (5). More recently, the National Institutes of Health (NIH) released new guidelines for grant submission that requires investigators to describe how they will “ensure the identity and validity of key biological resources,” including antibodies (NOT-OD-18-228). The international community has also come up with a proposal called Minimum Information About a Protein Affinity Reagent (MIAPAR), which aims to establish a stronger connection between antibody producers and users. MIAPAR-compliant data include information such as the production/purification process, experimental evidence, updated protocols, and other relevant details (7, 9).

In addition to improving the standardization of antibodies, antibody performance is another common source of variability in Western blotting. The Western blotting process relies on two key properties of primary antibodies: specificity, an antibody's ability to recognize and bind to its target antigen; and selectivity, an antibody's preference to bind its target antigen in the presence of a heterogeneous mixture of competing sample proteins. Well-characterized antibodies that consistently perform as expected are therefore essential for robust, reproducible research. Unfortunately, antibody performance can vary considerably between suppliers and even batches.

Antibody specificity and selectivity are highly dependent on the particular assay context and can be difficult to predict. Recommendations for antibody validation are different for each type of immunoassay, and an antibody that performs well in one assay, such as a Western blotting, might not be suitable for another assay (10–13). Even within one type of assay, small differences in assay conditions (intentional or unintentional) can affect antibody performance (8, 14–17). To ensure reproducible results, it is important to evaluate antibodies within the intended assays and experimental contexts. Assay-specific validation should confirm that the primary antibody is specific for its target antigen and that it selectively binds its target in the presence of other antigens. Here, antibody validation recommendations for Western blotting are discussed and outlined with relevant examples from the IWGAV guidelines.

## Results and discussion

### Defining antibody validation for Western blotting

**Figure 1.**
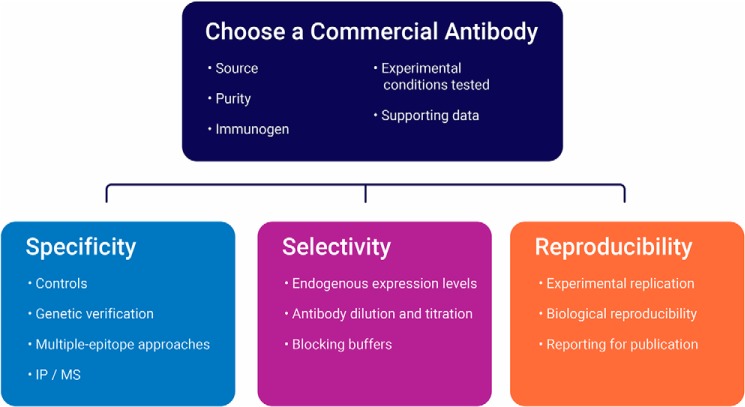
**Key elements of antibody validation.**

“Validation” is the experimental proof and documentation that a particular antibody is suitable for the intended assay or purpose (11). In a Western blotting context, proper validation is therefore proof that an antibody is specific to its intended target when bound to a membrane and can selectively bind to that target within a complex heterogeneous sample, such as cell or tissue lysates. Proposed methods of antibody validation for Western blotting include genetic controls, independent-epitope strategies, testing of multiple cell lines, proteomic approaches, additional evaluation of phospho-specific antibodies, and orthogonal or complementary methods (5, 18). Of these strategies, KO validation of antibodies is seen as the accepted “gold” standard for Western blotting and is increasingly being used by antibody vendors during the development and batch testing of primary antibody products. However, a single validation strategy is not sufficient, and a combination of strategies should be used for assay-specific validation of an antibody (18, 19), including those carried out by the antibody supplier/distributor. Fig. 1 demonstrates several practical approaches to verify the specificity, selectivity, and reproducibility of antibodies used in Western blot analysis.

#### 

##### Characterization by the supplier/distributor

When choosing an antibody for Western blotting experiments, suppliers/distributors should provide comprehensive information relating to the type of antibody and its performance in multiple applications. A datasheet for each antibody should include the source (polyclonal, monoclonal, or recombinant) and whether it has been purified, the type of immunogen used, lysate(s) tested, application-specific conditions (dilutions, sample concentrations, lot/batch information, etc.), and methods used to confirm antibody specificity and selectivity. Ideally, it should also provide validation and specificity data from multiple cell lines or tissues, with appropriate positive and negative controls. Selecting an antibody that has been validated for Western blot analysis is highly recommended. If the chosen suppliers/distributors provide validation data and assay conditions for Western blotting, this is an indication that they recognize the importance of thorough antibody characterization. Online search engines such as those listed in Table 1 offer advanced search and filtering options to identify commercial antibodies that may have user-submitted validation data, and scientists should be encouraged to share this type of data with the scientific community through appropriate portals.

**Table 1:** **Popular antibody search engines and directories**

**Search engines and directories**
www.antibodypedia.com/
www.antibodyresource.com
www.biocompare.com/Antibodies/
www.citeab.com/
www.linscottsdirectory.com/

##### Evaluation by the user: trust, but verify

**Figure 2.**
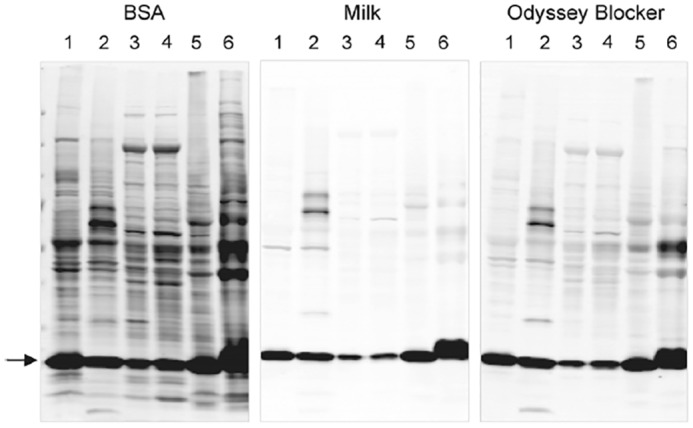
**Effect of blocking buffer on selectivity of an anti-cofilin primary antibody.** Cofilin (∼19 kDa) was detected on blots blocked with 5% BSA, 5% nonfat dry milk, or Odyssey blocking buffer. Blots were visualized with IRDye 800CW secondary antibody and laser-based digital imaging. All blots were processed identically and imaged together, with blocking buffer as the only variation. Tissue lysates are as follows: *lane 1*, mouse brain; *lane 2*, rat brain; *lane 3*, mouse liver; *lane 4*, rat liver; *lane 5*, mouse thymus; and *lane 6*, rat thymus. *Arrow* indicates the expected position of the 19-kDa cofilin band. Choice of blocking buffer greatly impacted the off-target binding of this antibody. For all primary antibodies tested in this study, BSA consistently produced more nonspecific bands than the other blocking buffers. Reprinted with permission from Ambroz *et al.* (62).

There is no guarantee that a given antibody will specifically detect and bind the target-of-interest, either in samples or in an experimental context (20), even if it has been raised against this target antigen. If the antibody has been validated for Western blotting either by the supplier/distributor or elsewhere, it should still also be confirmed by the user to be suitable for the intended experiment and assay context (18). Certain assay conditions, such as blocking reagents, can have a surprisingly large impact on antibody performance (Fig. 2). Each unique sample context is also relevant, given that samples from diverse sources and systems may contain different cross-reactive epitopes and produce different patterns of nonspecific binding.

##### Batch variation

A significant source of irreproducibility is variation between batches of antibodies. Vendors and researchers producing antibodies are highly encouraged to perform validation testing on every batch produced. Using recombinant antibodies eliminates the need for continued animal or hybridoma usage and reduces batch variation, especially when compared with polyclonal antibodies. Recombinant antibody production is carried out via a synthetic DNA expression vector introduced into a suitable expression system (21) that removes traditional reliance on hybridoma cells. This technique reliably produces high titers of homogenous antibody while avoiding hybridoma instability and/or the “genetic drift” that can compromise performance. The sequence for an antibody- variable domain can be accessed from a validated monoclonal-producing hybridoma, or from synthetic libraries through phage display technologies (22). Recombinant monoclonal antibodies provide the largest benefit to both manufacturers and scientists as they can be produced at scale in a short time with unlimited supply and greater consistency.

### Validating antibody specificity

**Figure 3.**
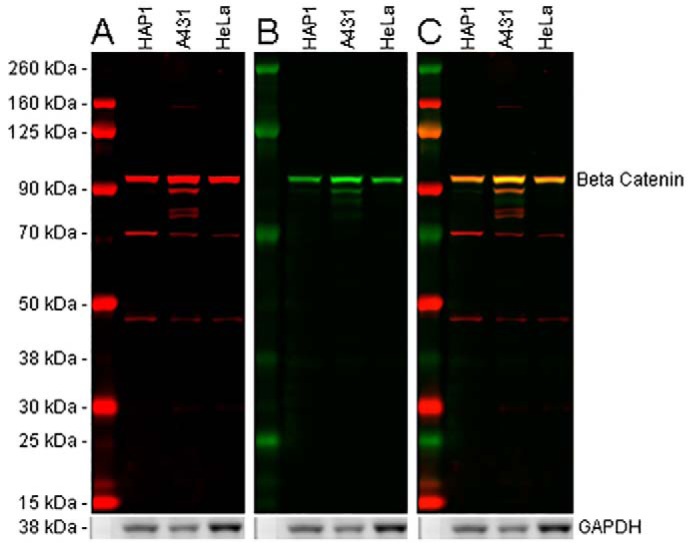
**Multiple epitope approach to detect β-catenin in cell lysates and to identify potential off-target antibody binding.** 20 μg of HAP1, A431, and HeLa lysates were loaded into a 4–12% BisTris gel and run under the MOPS buffer system. The membrane was blocked for 1 h using Odyssey blocking buffer (TBS) before incubation with mouse anti-β-catenin antibody (ab231305) (*A*) and rabbit anti-β-catenin antibody (ab35272) (*B*) and at a 1 μg/ml concentration and 1:5000 dilution (0.0000126 μg/ml), respectively. Antibody binding was detected using goat anti-rabbit IgG H&L (IRDye® 800CW) preadsorbed and goat anti-mouse IgG H&L (IRDye® 680RD) preadsorbed secondary antibodies at 1:20,000 dilution for 1 h at room temperature. *A,* ab231305, binding to the C terminus and visualized in the 700-nm channel (*red*), displays a strong band at 95 kDa. However, there are several bands at a lower molecular weight present in all lysates. *B,* ab32572, binding the N terminus of β-catenin and visualized in the 800-nm channel (*green*), displays a single band at 95 kDa with an additional faint band at 90 kDa in A431 lysate only. *C,* when both 800- and 700-nm channels are displayed, both ab32572 and ab231305 show a band at 95 kDa, identifying the full-length β-catenin protein. The additional bands seen for ab231305 are not clearly shown to overlay with ab32572. This could represent off-target binding or isoforms lacking the N-terminal binding domain for ab32572. Membranes were visualized using the Odyssey CLx imager with auto-intensity and 84-μm resolution. The membrane was then probed with an anti-GAPDH rabbit antibody conjugated to HRP (ab9385). Staining was developed using a GBOX XT-16 chemiluminescent imager with a 20-min exposure.

Detection of a single, distinct protein band of the expected molecular weight on a blot may not always indicate antibody specificity. Antibody specificity is the ability of an antibody to recognize and bind its target epitope. However, a single distinct band may represent the desired target protein, a cross-reactive sample protein, or a mixture of different proteins (23). By contrast, if a Western blotting shows multiple bands, this might not indicate nonspecific binding, as additional bands may represent protein degradation, post-translational modification (PTM) cleavage, splice variants of the target protein, or other proteins that also contain the target epitope. Therefore, it is important to confirm that the antibody recognizes the target protein in the intended assay and to understand the significance of any additional bands (Fig. 3) (5, 24).

### Controls

Appropriate positive and negative controls are essential for all Western blotting experiments. Controls aid the recognition of all potential sources of error and, if required, the need for intervention before results and interpretations are compromised (25, 26).

#### 

##### Positive controls

Positive controls provide information about the success of immunoblotting protocols. A positive result in a positive control lane indicates that the immunodetection protocol worked and lends validity to the other assay results. In contrast, a negative result for a positive control suggests that at least one step in a protocol did not work correctly or that the antibody used is responsible for the result.

Using lysate from cell lines known to express a specific target protein can provide a suitable positive control. With this type of control, a positive result indicates the protocol worked and that any negative results are potentially due to low expression or the absence of the target in the tested samples. “Overexpression” lysates can be appropriate positive controls for routine blots but should not be used to validate antibody selectivity or evaluate off-target binding (for details see under “Check confirming and Improving selectivity”).

Different cell lines and tissues will likely have varying protein compositions, so it is advised to test an antibody on multiple cell or tissue types to build up a protein expression profile. RNA and protein expression profiles can also be accessed online, making it easy to compare experimental data with expected cell line-/tissue-specific results (Table 2). It is critical to remember that biologically it is common for RNA levels to not match protein levels so direct comparisons between mRNA and protein levels may not be applicable (27, 28).

**Table 2:** **Online databases to check protein and RNA expression profiles**

Resource	Description	URL
Expression Atlas	Open access, gene and protein expression data across species and biological conditions (tissue/cell types, developmental stages, disease, etc.)	www.ebi.ac.uk/gxa/home
GeneCards®: The Human Gene Database	A searchable, comprehensive database of annotated/predicted human genes; integrates genomic, transcriptomic, proteomic, genetic, clinical, and functional data from many web sources	www.genecards.org
Cancer Cell Line Encyclopedia (CCLE)	A collaborative effort from Broad Institute and Novartis Institutes for Biomedical Research for genetic and pharmacological characterization of human cancer model	https://portals.broadinstitute.org/ccle
Human Protein Atlas	Open-source program; maps human proteins in cells, tissues, and organs using integrated omics technologies	www.proteinatlas.org

##### Negative controls

Negative controls provide information about antibody-binding specificity and can indicate problems such as off-target binding and false-positive results. Knockout (KO) and knockdown (KD) cell lines, lysates, or tissues can be very good negative controls but have some limitations (see under “Limitations”). If KO or KD samples are not available, the next recommended step is to consult one of the expression databases listed in Table 2. Protein expression scores based on antibody staining are indicative of the selectivity and specificity of the antibody used. Both parameters should be supported with published data from the literature where possible.

### Genetic verification

**Figure 4.**
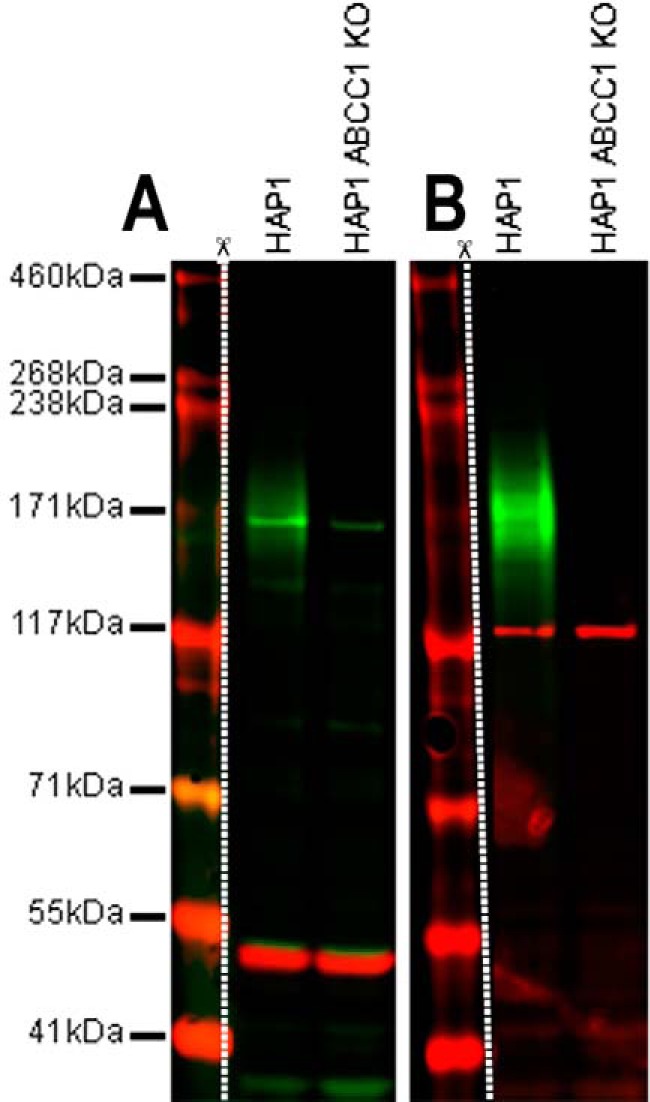
**Anti-MRP1 antibodies were tested against HAP1 WT and HAP1 ABCC1 KO samples in SDS-PAGE.** 20 μg of HAP1 (WT) and HAP1 MRP1 (ABCC1) KO were loaded into single 3–8% Tris-acetate gels and run under the Tris-acetate buffer system. The protein gel was transferred onto a single nitrocellulose membrane. Membrane was blocked in 3% milk (TBS + 0.1% Tween) solution before being spliced into two individual strips (*A* and *B*). *A,* membrane was incubated with mouse anti-MRP1 antibody (ab24102) and rabbit anti-α-tubulin antibody (ab52866) at a 1:20 dilution and 1:20,000 dilution, respectively. Antibody binding was detected using goat anti-mouse IgG H&L (IRDye® 800CW) preadsorbed and goat anti-rabbit IgG H&L (IRDye® 680RD) preadsorbed secondary antibodies at 1:20,000 dilution. ab24102 clearly displays a single band at 170 kDa in the 800-nm channel (*green*) that shows reduced signal in the HAP1 MRP-1 (ABCC1) knockout lysate. This confirms that ab24102 identifies MRP-1 as well as other off-target proteins. ab52866 staining α-tubulin at 50 kDa in the 680-nm channel (*red*) confirms equal protein loading across all lanes. *B,* membrane was incubated with anti-MRP1 rabbit antibody (ab233383) and mouse anti-vinculin antibody (ab130007) at a 1:1000 dilution and 1:20,000 dilution, respectively. Antibody binding was detected using goat anti-rabbit IgG H&L (IRDye® 800CW) preadsorbed and goat anti-mouse IgG H&L (IRDye® 680RD) preadsorbed secondary antibodies at 1:20,000 dilution. ab233383 displays the expected glycosylated smear for MRP1 between 150 and 200 kDa in the 800-nm channel (*green*) that is absent in the HAP1 MRP1 (ABCC1) KO lysate. This demonstrates that in HAP1 cells ab233383 reacts only with MRP1 (ABCC1). Ab130007 staining of vinculin at 125 kDa in the 700-nm channel (*red*) confirms equal protein loading across all lysates. Membranes were visualized using the Odyssey CLx imager with auto-intensity and 169-μm resolution. Separate images were required to visualize HiMark^TM^ pre-stained protein standard. *White dotted line* and *scissor symbol* denote splicing.

Removal of target protein expression within a given cell or tissue type generated by a gene KO technique is the accepted standard for validation of specificity by testing against a true negative sample. Signals are compared between positive control and knockout (negative control) samples, with the absence of the expected banding on a Western blotting in the KO sample strongly indicating primary antibody specificity (Fig. 4*B*). Reversely, a positive signal from validated KO lysates can be taken as evidence of antibody off-target binding and, consequently, poor specificity (Fig. 4*A*). An alternative to knockout validation is using transient gene KD. Both KO and KD have advantages and disadvantages, as discussed below. Because genetic verification can be performed in the relevant experimental context, it is an excellent tool for assay-specific validation of antibody performance. The increasing prevalence of CRISPR–Cas9-based KO techniques and resources has allowed many labs to genetically verify the specificity of commercially-available antibodies to key pharmacological targets. Studies using KO tests failed to confirm the specificity of commercial antibodies for G-protein–coupled receptors and α_1_-adrenergic receptors, etc. (75–78).

#### 

##### CRISPR–Cas9

A popular option for gene KO is the CRISPR/CRISPR–associated protein-9 (CRISPR–Cas9)-based strategy (29–33). CRISPR–Cas9 introduces site-specific dsDNA breaks at the target genetic location. The host cell DNA repair machinery may then disrupt the genetic locus through a frameshift mutation. These KO samples provide valuable tools for antibody validation, as a loss of signal during testing confirms that the tested antibody has bound to its intended target. The commercial availability of CRISPR constructs makes this technology accessible to most labs.

##### RNAi

KD by RNA interference (RNAi) is a useful alternative to KO for genetic verification. Small (or short) interfering RNAs (siRNAs) can induce transient silencing of the target gene and suppress target protein expression (34). On a Western blotting, siRNA KD can significantly decrease, or even completely suppress, the intensity of the target protein band, compared with the untreated sample. The Antibodypedia Validation Initiative recommends at least 50% KD of the target protein to serve as a valid negative control, compared with appropriate WT controls (35).

##### Limitations

1)Validation may not be suitable for target proteins that are essential for cell survival. 2) KD and KO may trigger compensatory changes in the physiology of the cell, leading to results that are not physiologically relevant (14). 3) Signal from an incomplete knockdown of target protein expression may mask off-target binding. 4) Although gene knockout results in complete and long-lived reduction in target protein abundance, the effects of gene knockdown are transient and dependent on consistent and sustained siRNA expression over time.

The extent of KD or KO must be validated experimentally before the sample is used as a control for antibody validation. This validation should include both genomic and proteomic approaches. Some gene-editing methods may not completely eliminate the targeted protein and/or result in a truncated protein, and a corresponding antibody may also detect the truncated forms of its target protein (16, 36–38).

In addition to using appropriate controls, often-overlooked aspects of the Western blotting process such as pre-adsorption (“blocking”) can also have significant effects on the outcome and consistency of experiments.

### Pre-adsorption with blocking peptides

Although pre-adsorption tests are not recommended for antibody validation, some researchers do use pre-adsorption controls in immunohistochemistry and other immunoassays (8, 14, 17–19, 39, 40). In this test, the antibody is pre-incubated with a molar excess of the immunogen used to generate it (the “blocking peptide”). If antibody binding is specific to its immunizing peptide, pre-adsorption will substantially decrease the intensity of protein staining. This method has several limitations, however. The blocking peptide may inhibit off-target binding of the antibody to antigens with the same epitope and produce an “illusion of specificity” (23), or the antibody may recognize related epitopes present in the sample. Although this test may prove that an antibody is specific, it cannot validate that it is selective (18, 41). Pre-adsorption controls may be most useful when using crude sera (23). As a monoclonal or affinity-purified polyclonal antibody has already been selected for its ability to bind the target antigen, pre-adsorption provides little additional information. Overall, routine use of pre-adsorption controls for antibody validation is discouraged (16, 18, 41, 42).

### Orthogonal and independent approaches

Orthogonal validation requires transcriptomics or antibody-independent proteomics to validate the differential protein expression seen with antibody assays. The transcriptomic analysis incorporates mRNA-based assays (43, 44) and gene-expression assays, such as luciferase reporters (45, 46), to determine whether sample gene expression correlates with antibody-binding patterns. Although these strategies can be used where endogenous protein expression is not well-characterized, they can be compromised by a lack of correlation between RNA expression and protein abundance (86), along with minimal variation in sample protein expression (47). Importantly, transcriptomic analysis will struggle to conclusively validate antibodies to post-translational modifications. This is because post-translational processing is often determined by cell signaling and protein interactions rather than gene expression. Proteomic orthogonal validation includes targeted proteomics and MS (48, 49). A high-throughput “capture Western blotting” that utilizes biotinylated protein samples and microsphere-based barcoded antibody assays (PAGE-MAP) has also recently been developed to aid validation using the IWGAV pillars (50). Although these are valuable tools for confirming antibody specificity through an analysis of protein levels between samples, they have issues differentiating co-migrating proteins of a similar molecular weight (71). However, these tools are most effective when combined with antibody assays, such as immunoprecipitation MS that can identify cross-reactive antigens through peptide mass fingerprinting (51).

Other independent validation approaches may include ELISA, immunohistochemistry, immunocytochemistry, tissue microarrays, and peptide arrays or reverse-phase protein arrays (5, 13). As discussed previously, an antibody used for Western blotting is not necessarily suitable for use in other applications. An independent antibody-based assay may therefore use a different antibody to the one undergoing Western blotting validation. Confirmation of Western blotting results via independent assays may provide biological verification or validation of an observed experimental effect. They can also support biological reproducibility via replicated results and/or independent verification of an observed experimental effect or response (26, 53). Importantly, findings of independent experimental approaches help to determine whether a finding is biologically relevant and is potentially applicable to other samples or experimental systems. It is important to consider the state of the proteins within the independent assay and the impact this may have on antigen recognition by the antibody. Formalin-fixed, paraffin-embedded tissue samples in immunohistochemistry may present a radically different range of epitopes when compared with denatured and reduced protein immobilized onto a membrane. Therefore, evidence of specificity within an independent assay can support, but not substitute, evidence of specificity within Western blotting experiments.

### Confirming and improving selectivity

#### 

##### Endogenous levels of target expression

**Figure 5.**
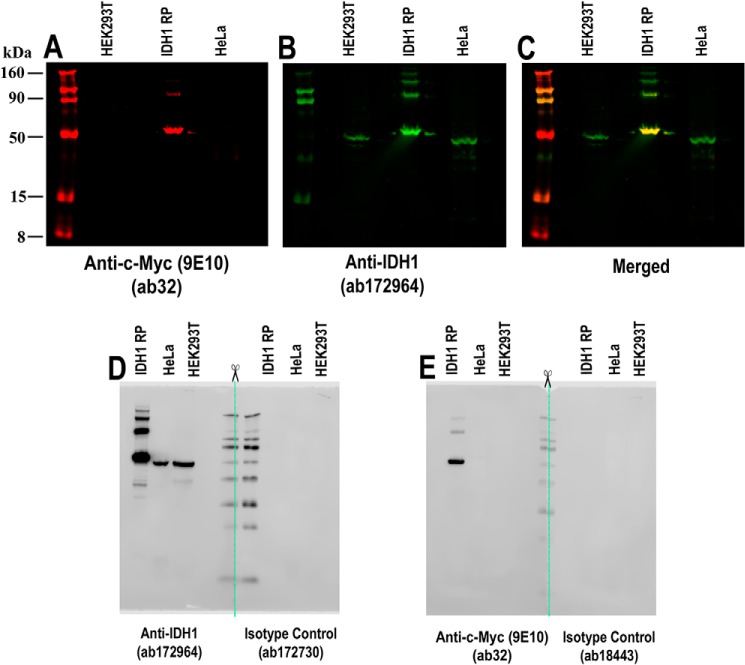
**Validation of IDH1 antibody using purified recombinant protein in multicolor and chemiluminescent Western blotting.** Multicolor and chemiluminescent Western blottings were performed using 10% Bis-Tris SDS-polyacrylamide gel and MOPS buffer system to validate the IDH1 antibody using a purified recombinant IDH1 protein (0.16 μg) containing a c-Myc tag in addition to HEK293T and HeLa whole-cell lysates. *A,* c-Myc protein tag present on the purified IDH1 recombinant protein is detected in the 700-nm channel (*red*) at 50 kDa via mouse anti-c-Myc antibody (ab32;1 μg/ml) using IRDye 680RD goat anti-mouse IgG (H + L) for detection. Some overspill of the recombinant protein into neighboring lanes is observed (*white box*). *B,* IDH1 recombinant protein and endogenous IDH1 protein, present in HEK293T and HeLa, is detected in the 800-nm channel (*green*) at 55 and 50 kDa, respectively, using rabbit anti-IDH1 antibody (ab172964; 1.2 μg/ml) and IRDye 800CW goat anti-mouse IgG (H + L) for detection. *C,* when both 700- and 800-nm channels are displayed, the signal from ab32 and ab172964 overlaps at 50 kDa, identifying the c-Myc–tagged IDH1 protein. No overlap is seen for the endogenous IDH1 present in HEK293T and HeLa whole-cell lysates. *A–C*, lysates loaded per lane are as follows: 20 μg of blocking buffer: Odyssey blocking buffer (TBS); imager: Odyssey® CLx; resolution: 169 μm; intensity: auto mode. Chameleon^TM^ Duo pre-stained protein ladder for accurate sizing of protein bands. *D,* single blot was split into two halves (*green line*) to be incubated with either rabbit anti-IDH1 antibody (ab172964; 0.115 μg/ml) or the corresponding rabbit monoclonal IgG isotype control (ab172730; 0.166 μg/ml) to detect the endogenous IDH1 protein present in HeLa and HEK293T as well IDH1 recombinant protein. Both halves were incubated with HRP-conjugated goat anti-mouse IgG (H + L). *E,* single blot was split into two halves (*green line*) to be incubated with either mouse anti-c-Myc antibody (ab32; 1 μg/ml) or the corresponding mouse monoclonal IgG1 isotype control (ab18443; 1 μg/ml) to detect c-Myc protein tag present on the purified IDH1 recombinant protein but absent in HEK293T and HeLa whole-cell lysates. Both halves were incubated with HRP-conjugated goat anti-rabbit IgG (H + L). Blots were detected with WesternSure® PREMIUM chemiluminescent substrate (LI-COR 926–95000) and imaged on an Odyssey® Fc with the following resolution: 125 μm and exposure of 2 min. Lysate loaded per lane: 20 μg; protein ladder: WesternSure® pre-stained chemiluminescent protein ladder (LI-COR 926-980000); blocking buffer: intercept blocking buffer (TBS); intercept T20 (TBS) antibody diluent.

In a Western blotting experiment, the ratio of the target to other proteins in the sample is skewed, and unrelated sample proteins are typically present in considerable excess. The antibody must be able to locate and selectively bind the target antigen in a complex mixture. Target protein concentration strongly influences antibody selectivity, and therefore purified or overexpressed target protein should not be used as the sole protein sample for antibody validation. Overexpression of the target alters the balance of protein abundance in assays, giving the antibody an artificial advantage (5, 54, 55). An overexpressed target may mask off-target binding that creates false-positive results at endogenous target expression levels. If an affinity-tagged protein is used for validation it should be expressed at endogenous levels (5). Purified protein gives no opportunity to evaluate off-target binding but can be valuable for confirming antibody reactivity with the target. Nevertheless, endogenous samples should always be present to evaluate potential off-target binding (Fig. 5) for proper demonstration of antibody specificity.

##### Antibody dilution and concentration

Antibody concentration and incubation time can affect selectivity and nonspecific binding (11, 56). A high-affinity primary antibody can bind and detect its antigen at a low working concentration, whereas a low-affinity antibody will require a higher working concentration. By contrast, higher working concentrations increase the likelihood of off-target binding and cross-reactive bands that could mask the protein of interest. The optimal concentration of the primary antibody that produces the best blot signal–to–noise ratio should be determined experimentally. A product's recommended usage conditions can be taken as guidelines; however, they may be based on different assay conditions or reagents. A titration experiment with a series of antibody dilutions is a practical way to do this (Table 3 has guidelines on the dilution of unpurified antibodies).

**Table 3:** **Dilution guidelines for unpurified antibody preparations** Unpurified antibodies will not have a concentration stated on the vendor datasheet. For most whole antisera, culture supernatants, or ascites fluid products, the concentration may be unknown. Unpurified antibody preparations vary significantly in specific antibody concentrations. If the specific antibody concentration of an unpurified antibody preparation is unknown, these concentration estimates may be used as a rough guideline. Please remember that these dilutions and concentration estimates are only a starting point, and dilutions may need to be adjusted based on the experimental results. IHC, immunohistochemistry; ICC, immunocytochemistry; EIA, enzyme immunoassay. NA, not applicable.

Assay	Tissue culture supernatant	Ascites	Whole antiserum	Purified antibody
Western blot/dot blot	1:100	1:1000	1:500	1 μg/ml
IHC/ICC	Neat: 1:10	1:100	1:50–1:100	5 μg/ml
EIA/ELISA	1:1000	1:10,000	1:500	0.1 μg/ml
FACS/flow cytometry	1:100	1:1000	1:500	1 μg/ml
Immunoprecipitation	NA	1:100	1:50–1:100	1–10 μg/ml
Concentration estimate	1–3 mg/ml	5–10 mg/ml	1–10 mg/ml	NA

For the best results, it is good practice to maintain consistent experimental conditions by choosing a fixed incubation time with a consistent secondary antibody concentration and to test each primary antibody dilution on the same type of sample. Secondary antibody concentration also affects the selectivity of a primary antibody. Too much secondary antibody will increase off-target binding on a blot, and too little may produce faint signals. Note that the recommended dilution range depends on the type of secondary antibody conjugate and detection method selected. For example, the recommended dilution for an HRP conjugate and chemiluminescent detection may not be appropriate for a fluorescent antibody conjugate.

Monoclonal antibodies have similar batch–to–batch consistency, and it may only be necessary to perform the titration once. However, especially for polyclonal antibodies, there can be differences between batches of the same antibody, and it is good practice to perform a titration for each new batch. Because antibody concentration may vary between batches and/or product suppliers, it is essential that both the 1) dilution factor and 2) antibody concentration in mass units per volume of diluent (μg/ml) are reported in publications (17). Storage and re-use (“recycling”) of diluted primary antibodies is not recommended as the inconsistent quality, titer, and stability of recycled antibodies is a potential major source of error that can negatively affect the reproducibility of results.

##### Blocking buffers

A blocking buffer should reduce the nonspecific binding of antibodies to off-target sample proteins on the membrane and to the membrane itself. It should also stabilize the blotted sample proteins without disrupting their retention on the membrane surface (57–59). This enhances the specificity and selectivity of the antibody, increasing the signal–to–noise ratio for detection of the target.

Blocking buffers should not mask the interaction of the antibody with the target antigen or display enzymatic activity or other properties that may interfere with antibody–antigen interaction or target protein detection (57). Bovine serum albumin (BSA), nonfat dry milk, and casein are commonly-used blocking buffers that differ in blocking strength and may interfere with target protein detection in some assay contexts.

Nonfat dry milk is inexpensive and widely used. It has a high blocking strength that may disguise some antigens and can also inhibit biotin–streptavidin interactions (60). Endogenous biotin and IgGs in milk can also potentially cross-react with sheep or goat secondary antibodies. Casein, an abundant protein in found in nonfat dry milk, is a phosphoprotein that may cross-react with some phospho-specific antibodies and increase background signal (56).

BSA is a popular alternative to milk-based blockers and may be more suitable for detection of antibodies labeled with biotin or alkaline phosphatase (57, 59). However, some sources of BSA contain phosphotyrosine residues that may bind anti-phosphotyrosine antibodies, and endogenous carbohydrates may interfere with detection of lectins (57, 59). BSA blockers can increase nonspecific banding. When used for fluorescent Western blotting assays, BSA can cause increased membrane background (Fig. 2) (61, 62). Nonmammalian blocking agents are less likely to contain cross-reactive epitopes and may produce lower background signal than mammalian reagents, which are often available from suppliers of fluorescent Western blotting platforms.

Extended blocking times and/or high concentrations of blocking agents can mask antibody–antigen interactions, reducing the signal intensity of target protein bands (56, 58). Excessive blocking can cause loss of sample proteins from the surface of the membrane, particularly with nonfat dry milk. Den Hollander and Befus (63) demonstrated nonselective elution of blotted proteins from the membrane in proportion to the amount of milk used, with progressive loss over time. The strength of blocking agent and length of incubation should be considered when staining for low abundance proteins.

Membrane blocking, antibody incubations, and washes often use Tris-buffered saline (TBS) or phosphate-buffered saline (PBS) buffer systems. TBS buffers should be used for the detection of phosphoproteins, in order to avoid interference from the phosphates in PBS buffers. TBS can also be used for the detection of alkaline phosphatase–conjugated secondary antibodies (58). In some cases, a product supplier might specifically recommend PBS or TBS buffers for an antibody to obtain optimal results.

##### Experimental replication

At a minimum, results should be repeatable within and between Western blotting experiments (18, 64), and all samples should be run with replicates. A robust antibody should generate the same result regardless of the day, the user, or the laboratory with replicates (both biological and technical) of *n* ≥ 3 as the norm rather than the exception. Replication of results in a different laboratory adds another level of confidence. It confirms the robustness of the antibody and the experiment itself, given the multitude of small differences that may exist between the methods, equipment, and reagents used (64).

Operator habits and technique at the bench can affect results, with as much as ∼80% of variation arising from “inter-operator” variability (65). Such unintentional variations such as minor differences in antibody dilution, antibody incubations, washes, and other aspects of the Western blotting procedure can be a substantial source of error. Detailed reporting of experimental procedures is an effective way to reduce this type of error, with many peer-reviewed journals now encouraging this level of transparency.

Even so, it is still common for published studies to omit key details of the Western blotting procedure such as the type of detection performed (enzymatic *versus* fluorescence) and how the authors document results (film exposure, timing of exposure, digital imaging, etc.). Without such critical information, other researchers may be unable to reproduce the experimental results.

Even so, it is still common for published studies to omit key details of the Western blotting procedure used. Such information includes the system on which fluorescent detection was captured and the associated system settings. Similarly, for enzymatic detection it is important to report whether film or camera was used and what the exposure time was. Without such critical information, other researchers may be unable to reproduce the experimental results.

Tackling the current challenges and considerations in traditional Western blotting techniques is important, as is the need to look forward to other techniques and variations on this technique that will be of significant benefit to researchers in the future. Some of these techniques may improve a number of parameters that are current challenges to the traditional technique and therefore could promote greater consistency of results, including multiplexing, advances in expression tags, and the detection of protein modifications.

## The future is now: Multiplex fluorescent Western blot target detection

**Figure 6.**
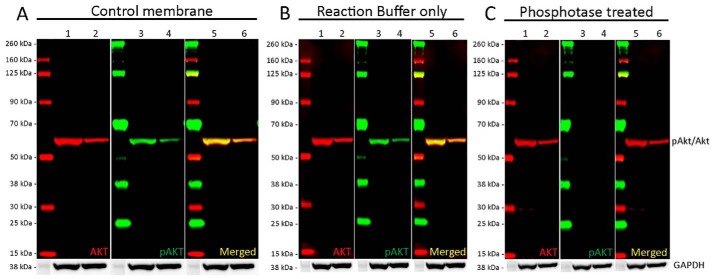
**Analysis of phosphorylation specificity using multiplexed Western blotting and alkaline phosphatase membrane treatment.** 20 μg of LNCaP (*lanes 1, 3,* and *5*) and Jurkat (*lanes 2, 4,* and *6*) whole-cell lysates were loaded into a 4–12% Bis-Tris gel and run under the MOPS buffer system. Following transfer, membranes were cut and separated for alkaline phosphatase treatment. Membranes were blocked for an hour using Odyssey Blocking Buffer (TBS) before incubation with rabbit anti-AKT1 (serine 473) antibody (ab81283) and mouse anti-AKT1 (ab108202) antibody at a 1:2000 dilution (9.7 μg/ml) and 1:500 dilution (1.67 μg/ml), respectively. Antibody binding was detected using goat anti-rabbit IgG H&L (IRDye® 800CW) preadsorbed and goat anti-mouse IgG H&L (IRDye® 680RD) preadsorbed secondary antibodies at 1:20,000 dilution. *A,* in the untreated control membrane, ab108202 (*red*) clearly recognizes a single band at 60 kDa in both lysates that corresponds with the molecular weight of AKT1. ab81283 (*green*) also detects a single band at 60 kDa in both lysates corresponding the phosphorylated form of AKT1. Both bands clearly overlap when the channels are merged. *B,* membranes treated with the alkaline phosphate reaction buffer displayed identical banding to the control membranes for both AKT1 (ab108202, *red*) and for pAKT1 (ab81283, *green*). C. ab108202 (*red*) displays the expected band at 60 kDa for AKT1. No signal is seen for ab81283 (*green*). Membranes were visualized using the Odyssey CLx imager with auto-intensity and 84 μm resolution. Membranes were then probed with an anti-GAPDH rabbit antibody conjugated to HRP (ab9385). Staining was developed for 20 min using a GBOX XT-16 chemiluminescent imager.

**Figure 7.**
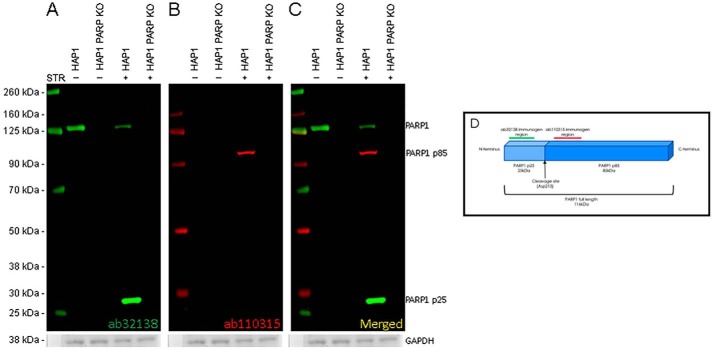
**Analysis of PARP1 cleavage by Western blotting.** HAP1 WT and HAP1 PARP1 KO cells with (+) and without (−) staurosporine (*STR*) treatment were lysed, and 20 μg of total protein was loaded into a 4–12% Bis-Tris gel and run under the MOPS buffer system. The membrane was blocked for an hour using Odyssey blocking buffer (TBS) before incubation with rabbit anti-PARP1 antibody (ab32138) and mouse anti-cleaved PARP1 ab110315 at a 1:1000 dilution (9.7 μg/ml) and 1 μg/ml concentration, respectively. Antibody binding was detected using goat anti-rabbit IgG H&L (IRDye® 800CW) preadsorbed and goat anti-mouse IgG H&L (IRDye® 680RD) preadsorbed secondary antibodies at 1:20,000 dilution. *A,* full-length PARP1 was identified at 130 kDa by ab32138 (*green*) in HAP1 WT untreated lysates. Following treatment with staurosporine and cleavage of PARP1 in HAP1 WT cells, ab32138 detects a significantly weaker full-length PARP1 signal at 130 kDa alongside a new, stronger band at 28 kDa that represents the N-terminal cleavage product. No banding at either molecular weight is seen in treated or control HAP1 PARP1 knockout lysates. *B,* following treatment with staurosporine and cleavage of PARP1 in HAP1 WT cells, ab110315 (*red*) identifies the C-terminal cleavage product of PARP1 at 100 kDa. No banding is seen in the untreated WT control or in HAP1 PARP1 knockout lysates. *C,* overlay of both 800 and 70 nm displays clear identification of full-length PARP1 and cleavage products in staurosporine-treated HAP1 cells. Membranes were visualized using the Odyssey CLx imager with auto-intensity and 84-μm resolution. The membrane was then probed with an anti-GAPDH rabbit antibody conjugated to HRP (ab9385). Staining was developed for 20 min using GBOX XT-16 chemiluminescent imager. *D,* illustration of full-length PARP1 protein and associated cleavage products. Immunogen domains of ab32138 (*green*) and ab110315 (*red*) are displayed with the corresponding imaging channel color.

The future of Western blotting lies in harnessing the biological sample to the maximum capacity via multiplexed detection. Although Western blotting is a staple assay of many labs, they still present several limitations. The assay can be manual, slow, and time-intensive during most steps and difficult to automate at scale. Electroblotting to a membrane and subsequent antibody incubations can also lead to substantial antigen loss (50). Capillary electrophoresis and microfluidic Western blotting (66) represent two areas of innovation of the core assay concept. Microfluidic Western blotting improves on the current process by both down-scaling size and increasing multiplexing, thus achieving detection of 10 separate proteins on one membrane with a fraction of the sample (67, 68). Capillary Western blotting through capillary gel electrophoresis (CGE) replaces the existing gel and membrane system with protein separation, immobilization, and staining in a single column (69). CGE is considered to increase reproducibility of Western blottings, improve quantification, reduce hands-on time, and improve automation potential (70). It is important to note that with the change in assay context, the behavior of antibodies also changes. Therefore, direct comparison of protein expression and modification on a single membrane by multiplexing antibodies provides the best advancement in antibody validation for Western blotting. Spectrally-distinct fluorophores can be conjugated to primary antibodies or secondary detection antibodies to allow simultaneous detection on one blot. Fig. 3 demonstrates how co-localization and co-migration of independent epitopes in the same target band can be evidence of antibody specificity. Here, domain-specific primary antibodies directed against the N and C termini of the target are used to analyze the overlap of staining and domain-specific bands. This technique can also be used to analyze protein isoforms (20, 71). This method also enables analysis of protein modifications relative to the unmodified target. Fig. 6 demonstrates analysis of phosphorylation of Akt, and Fig. 7 shows proteolytic cleavage of PARP1, with both figures analyzing the endogenous, unmodified protein within the same samples. Relative quantification of ubiquitination, sumoylation, glycosylation and acetylation are obtainable in this system (53–56). Current methods rely on detection of primary antibodies using spectrally-distinct fluorophores, either conjugated directly to the primary antibody or through binding of secondary antibodies. Digital detection of fluorophores can also be used in combination with enzymatic detection (Figs. 5–7), further increasing multiplex capabilities.

### Multiplexed detection of Expression tags

Expression tags enable a different kind of multiple-antibody strategy. In this approach, the target protein can be modified with an affinity tag (such as His_6_, c-Myc, HA, or FLAG) or fluorescent protein (GFP, RFP, mCherry, etc.). The antibody under evaluation and a well-characterized immunoreagent that binds the tag are both used to examine a panel of samples on separate blots. Correlation of results between the two blots is an indicator of antibody specificity. Although this approach can be taken with traditional Western blotting techniques, Fig. 5 demonstrates the advantages of multiplex fluorescence Western blotting when performing multiple-antibody analysis. Both techniques demonstrate co-localization of primary antibody binding and detection of the epitope tag; however, multiplexing and visualization of binding on the same blot remove potential errors between membranes and ensures greater accuracy of analysis. When planning to use tagged proteins, there are some potential issues to be addressed. An affinity tag may affect the conformation, trafficking, or function of the target protein (72–75). This risk can be minimized by placing the tag at the N or C terminus of the target protein. In rare instances, the junction of the target protein and expression tag may create a “neoantigen” that the antibody cannot recognize (72–74).

### Validation of multiplex immunoblotting

The binding of both primary and secondary antibodies should be validated in a multiplex format to ensure that cross-reactivity does not affect the interpretation of experimental results. Primary antibodies should have been previously validated, with a well-characterized binding pattern under similar experimental conditions. It is important to run control blots with separate and combined primary antibodies to identify potential cross-reactivity or binding interference. Because of this, it is important to be able to discriminate between primary antibodies on a single blot. When using secondary antibodies, this can be achieved through the recognition of different antibody species or isotypes. Alternatively, primaries can be conjugated to different fluorophores or enzymes for direct detection. Detection of new cross-reactive bands may indicate that the assay requires further optimization or that a different primary antibody might be required.

Secondary antibodies should be validated across all species or isotypes present within the immunoassay. To control for secondary cross-reactivity, each primary antibody should be tested in combination with the opposite secondary antibody of mismatched specificity and checked for the presence of spurious bands. Highly cross-adsorbed secondary antibodies can prevent cross-reactive binding to other antibody species used in the assay. This type of control is particularly important in certain circumstances, for example where two protein targets co-migrate closely or the experimental samples contain Igs that could cross-react with the secondary antibodies.

### Validation of pan/phospho and pan/post-translational modification analysis

PTMs such as protein phosphorylation can be analyzed by multiplexing a modification-specific primary antibody with an antibody that recognizes the target protein regardless of its modification state (pan-specific antibody). Multiplexed analysis allows the monitoring of relative changes in phosphorylation levels across a group of samples, with the target protein acting as its own internal loading control. This approach eliminates the error and uncertainty introduced by stripping and re-probing of blots (76) and accounts for changes in abundance of the target protein.

The *Journal of Biological Chemistry* now recommends the above method for quantitative Western blot analysis of phosphorylation, stating that “signals obtained using antibodies specific for phosphorylated epitopes should be normalized to the total protein level of the target protein” (Instructions for Authors (2018) JBC: http://www.jbc.org/site/misc/ifora.xhtml (accessed March 16, 2018)). However, this method only compares the relative abundance of modified forms and does not indicate stoichiometry (76).

Validation of antibody specificity is crucial for pan/PTM analysis, as the modified and unmodified forms of the target are likely to co-migrate closely. Therefore, the following key validation steps below should be kept in mind. 1) Perform separate Western blottings with each primary antibody to characterize the banding patterns. 2) Test each primary antibody separately with the opposite, mismatched secondary antibody as described above. Cross-reactive bands could affect data analysis and interpretation if modified and unmodified forms of the target co-migrate or multiple forms are detected on the blot. 3) Verify that the PTM-directed antibody is specific for the intended modification and does not cross-react with the unmodified target. Enzymatic addition or removal (Fig. 6) of the PTM is an elegant validation tool for this. 4) Evaluate any new pan- and PTM-specific antibody combination for possible binding interference. In one study, single-plex and multiplex antibody combinations were examined; however, little to no interference effect was observed (77).

### Reporting Western blotting data in peer-reviewed publications

Antibody validation is a complex problem and is currently without completely clear or established international standards. However, each researcher can contribute to the solution by including the full details of validation and experimental methods in manuscript submissions, providing raw validation data when requested, depositing validation results and methods in existing databases, and providing feedback to product suppliers about purchased antibodies (24, 78).

Detailed disclosure of antibody-related validation details and experimental methodologies is an important first step, and some journals now require or strongly encourage this practice (79–81). Recommendations for the reporting of antibody validation for Western blotting are summarized in Table 4. Detailed information for all antibodies should be included, including the species of origin, supplier name, catalogue number, clone name or number, batch or lot number, antibody name, preparation method (*i.e.* monoclonal, polyclonal, recombinant), type of antigen used, and research resource identification (RRID) (82), or other unique identifiers (7, 82–84).

**Table 4:** **Reporting antibody validation results for Western blotting**

**Antibody details**
Vendor/supplier
Antibody name
Catalogue and clone numbers
Species of origin
Immunogen
Lot or batch number
Research Resource Identification (RRID) (if available)
**Validation strategy**
Genetic, orthogonal, and/or other verification
Positive and negative controls
Antibody titration and optimization
Replication
**Samples**
Source (*e.g.* organism, model, cell type, or line)
Endogenous, purified, tagged, or overexpressed target protein
Dose, time, activator/inhibitor, or other treatment conditions
Sample preparation (*e.g.* lysis conditions, no. of cells)
**Validation methods**
Amount of sample loaded
Western blotting conditions (including blocking reagent, primary and secondary antibody concentrations, and incubation times)
Detection/visualization methods

Any evidence of primary antibody validation and specificity should be reported, and if possible raw data for validation experiments should be included to facilitate peer review. When reporting validation information, include the antibody characteristics tested, validation strategy and methods, assay conditions, including blocking buffers, antibody dilutions, and incubation times, positive and negative controls used, and sample information, including cell or tissue type, organism/species, lysate, purified or overexpressed protein, and growth or stimulation conditions (24, 78). Adopting thorough and consistent publication practices is essential for transparency, validation, and reporting with research antibodies.

## Conclusion

Antibodies are a major source of variation in Western blotting results and subsequent analysis. Inconsistencies in the quality, validation, and reporting of research antibodies have previously been demonstrated to compromise the reproducibility of research results (3, 7, 85, 86). In this paper, practical recommendations have been outlined that can help researchers best choose, validate, and use research antibodies in Western blotting assays. We have adopted the five pillars of antibody validation proposed by the IWGAV (5), specifically, the enhanced strategies proposed recently for Western blotting applications (87). The data presented here demonstrate the use of a multipronged approach for antibody validation, including genetic knockout (Fig. 4), independent antibodies (Fig. 3), and recombinant expression strategy (Fig. 5). Although unintended variation cannot be eliminated, it is important to verify antibody performance, especially in the relevant experimental context and assay. Emphasis has been placed on strategies for Western blotting validation in Table 5.

**Table 5:** **Suggested benchmarks for user validation of antibody performance in Western blotting**

**Specificity**
✓Verify by knockdown/knockout of target expression
✓ Appropriate positive and negative controls
**Selectivity**
✓ Detect target at endogenous levels in a complex sample
✓ Optimize antibody dilutions, assay conditions
**Reproducibility**
✓ Repeat/reproduce the experimental result
✓ Confirm observed effect with a complementary method

Collectively, the reproducibility of research antibodies is a high priority in the scientific community. Although a universal framework of standards has not yet emerged, there is general agreement that rigorous validation should include, at a minimum, evaluation of antibody specificity and reproducibility (88–90). However, the requirements and recommendations for antibody validation are different for each type of immunoassay, and antibody reagents must be evaluated for the specific assay and context in which they will be used.

**Figure 8.**
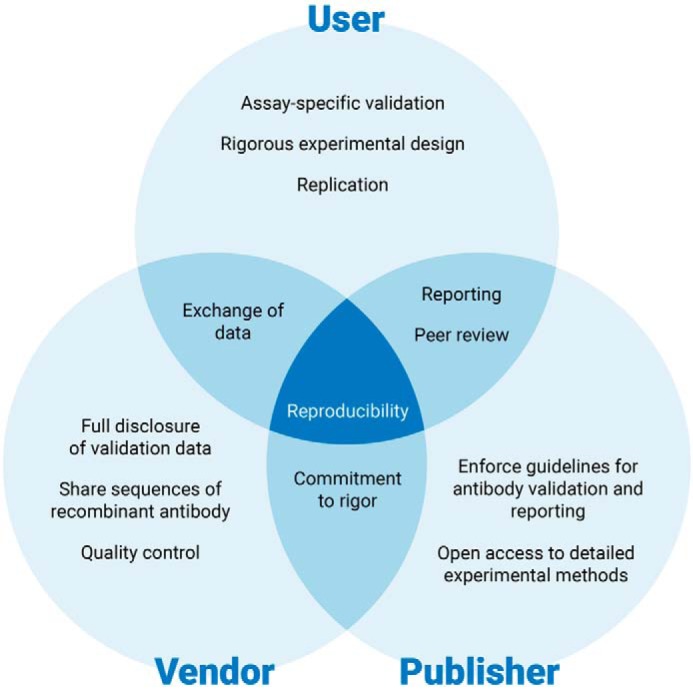
**Collaboration and cooperation are required to address the antibody reproducibility problem.** Reproducibility requires the combined efforts of the user, vendor, and publisher. Users should perform assay-specific validation of antibody performance and conduct well-designed experiments. Validation results, whether good or bad, can be openly shared and detailed methods reported when the scientific findings are published. The vendor's role is to provide high-quality, well-characterized antibodies with detailed disclosure of methods and results. The publisher can formulate and enforce guidelines for antibody validation and data reporting, providing access to detailed methods and other supporting information. Researchers must work together to standardize the way research antibodies are validated, used, and reported.

Having checkpoints at each stage of antibody production, sale, transport, usage, and publication of the data generated using antibodies are some of the most effective measures that can be used by the life sciences industry to counter the reproducibility crisis in Life Science and Biomedical Research. Suppliers/distributors, scientists, journals, and funding agencies need to have a consensus and detailed processes and protocols on antibody validation. This is a very exciting time for techniques such as Western blotting with the availability and accessibility of newer technologies like recombinant antibodies, multiplex-compatible fluorophores, capillary gel electrophoresis, and microfluidic Western blotting all highlighting the importance of antibody-based protein detection assays. Antibody suppliers and publishers should also continue to refine and expand their commitment to rigorous, reproducible science and provide corresponding quality control and validation data alongside corresponding developments in validation-enabling technologies. If antibody suppliers and consumers can work together toward this shared goal (Fig. 8), it might be possible to overcome the current antibody reproducibility crisis and enter a more rigorous, productive era of antibody-based research.

## Materials and methods

### Cell culture

A431 (ECACC; 85090402), HeLa (ECACC; 93021013), LNCaP (ATCC; CRL-1740^TM^), Jurkat (ATCC; TIB-152^TM^), human near-haploid cells (HAP1) (Horizon Discovery, Cambridge, UK; C631), HAP1 PARP1 knockout (Horizon Discovery; HZGHC003943c006), A549 and A549 ABCC1 knockout cells (Oxford Genetics, Oxford, UK) were cultured at 37 °C in a humidified atmosphere containing 5% CO_2_. A431 and HeLa cells were cultured in minimum essential media (Life Technologies, Inc.; 21090022) supplemented with 10% fetal bovine serum (FBS) (Life Technologies, Inc.; 10101145), 2 mm l-glutamine (Life Technologies, Inc.; 25030-024), and 1% nonessential amino acids (Life Technologies, Inc.; 11140-050). LNCaP and Jurkat were cultured in RPMI 1640 media (Life Technologies, Inc.; 22409-015) supplemented with 10% FBS and 2 mm l-glutamine. A549 and A549 ABCC1 KO cells were cultured in Dulbecco's modified essential media (Life Technologies, Inc.) supplemented with 5% FBS and 2% l-glutamine. Cells were cultured up to 80% confluence and pelleted before being stored at −80 °C.

### Cell lysis

Cells were resuspended in 150 μl of radioimmunoprecipitation assay (RIPA) buffer (Sigma; R0278) supplemented with 10× protease inhibitor mixture (Sigma; P2714) and 10 μl of phosphatase inhibitor mixture (Sigma; P5726) per 1 × 10^7^ cells and incubated on ice for 10 min. Lysates were sonicated for 50-s intervals until no pellet was visible and then clarified by centrifugation at 8000 relative centrifugal force for 10 min at 4 °C. The supernatant was taken and protein concentration determined by bicinchoninic acid assay, as described previously (52). Lysates were diluted in 4× SDS loading buffer (Invitrogen; NP0007) and 10% 1 m DTT before being stored at −80 °C.

### Commercial cell lysates

Isocitrate dehydrogenase (IDH1) (NM_005896) human recombinant protein (OriGene no. TP310582), IDH1 (NM_005896) human overexpression lysate supplied with parental HEK293T lysate (OriGene no. LY401782; HEK293T LY500001; lot no. 0076CF), and IDH1 knockout cell lysate (supplied with parental HeLa control lysate) (Origene no. LC810112, LC810Hela; lot no. 1601) were mixed with either 2× protein loading buffer (PLB) (LICOR no. 928-40004) or 2× SDS buffer (OriGene) and denatured by boiling at 97 °C for 5 min. The aliquots were then immediately placed on ice for 5 min before being spun down.

### Western blotting

Lysates were loaded onto NuPAGE 4–12% 15-well Bis-Tris gels (Invitrogen; NP0323BOX), NuPAGE^TM^ 10% Bis-Tris, 1.0 mm, 10-well protein gels (ThermoFisher Scientific; NP0301BOX), or NuPAGE 3–8% Tris acetate gels (Invitrogen; EA03752BOX) and run at 200 V for 55 min or 150 V for 60 min, respectively. Gels were wet transferred using 20% methanol onto either NuPAGE nitrocellulose 0.2-μm pore sheets (Invitrogen; LC2000) or Odyssey® nitrocellulose membranes, 0.22 μm, 7 × 8.5 cm **(**P/N 926-31090). Membranes were blocked for 1 h at room temperature in Odyssey Blocking Buffer (TBS) (LI-COR 927-50000) or the stated blocking solution. Primary antibodies were diluted with their respective blocking buffers (see figure legends) and incubated overnight at 4 °C. Washes were performed with TBS 0.1% Tween 20 (TBS-T) before addition of secondary antibody for 1 h at room temperature. Washes were performed with 1× TBST before imaging. For demonstration of equal protein loading following multicolor imaging, membranes were incubated with HRP-conjugated GAPDH primary antibody (ab9385) for 1 h at room temperature in Odyssey Blocking Buffer (TBS). Membranes were washed with TBS-T before staining was developed using Optiblot ECL Detect kit (ab133406) and imaged on a GBOX XT-16 chemiluminescent imager with a 20-min exposure.

### Membrane alkaline phosphatase treatment

Following the Western blotting transfer step, membranes were rinsed with deionized water before incubation with FastAP Thermosensitive Alkaline Phosphatase (ThermoFisher Scientific; EF0652), at 150 units/ml in the supplied FastAP buffer, for 1 h at 37 °C. Incubated membranes were agitated every 15 min. Control membranes were incubated in FastAP buffer only. Membranes were rinsed twice with TBS 0.1% Tween 20 (TBS-T) before being washed for 10 min. Membranes were then incubated in the required blocking buffer

### Antibodies

The following primary antibodies used in this study were purchased from Abcam (Cambridge, UK): anti-MRP1 (ab24102), anti-α-tubulin (ab52866), anti-MRP1 (ab233383), anti-vinculin (ab130007), anti-IDH1 (ab172964), anti-c-Myc tag (ab32), rabbit monoclonal IgG isotype control (ab172730), mouse monoclonal IgG1 isotype control (ab18443), anti-β-catenin (ab231305 and ab35272), anti-GAPDH HRP-conjugated (ab9385), anti-PARP1 (ab32138), anti-cleaved PARP1 (ab110315), anti-AKT1 pSer-473 (ab81283), and anti-AKT1 (ab108202). Antibody binding for multicolor Western blotting was detected using goat anti-rabbit IgG H&L (IRDye® 800CW), goat anti-mouse IgG H&L (IRDye® 680RD), goat anti-mouse IgG H&L (IRDye® 800CW), and goat anti-rabbit IgG H&L (IRDye® 680RD) preadsorbed secondary antibodies (LI-COR Biosciences; 926-32211, 926-68070, 926-32210, and 926-68071). Chemiluminescent detection was achieved using HRP-conjugated goat anti-mouse IgG (H + L) (LI-COR 926-80011; lot no. C80716-02) and HRP-conjugated goat anti-rabbit IgG (H + L) (LI-COR 926-95000; lot no. C90214-01).

### Imaging

Multicolor Western blots were imaged wet on the Odyssey® CLx imaging system using 700- and 800-nm channels and visualized using ImageStudio software version 5.2 (LI-COR Biosciences). HRP-conjugated GAPDH primary antibody binding was detected using a GBOX XT-16 chemiluminescent imager (Syngene). Chemiluminescent detection of IDH1, c-Myc, and isotype controls was imaged with Odyssey Fc® imaging system. Adobe Photoshop Elements 5.0 and 13.0 was used to prepare image panels and annotations.

## Author contributions

L. P.-K., S. H., A. S., and A. R. S.-G. conceptualization; L. P.-K. and S. H. resources; L. P.-K., S. H., and A. C. R. data curation; L. P.-K. and S. H. software; L. P.-K., S. H., and S. D. S. formal analysis; L. P.-K. and S. D. S. supervision; L. P.-K. and S. D. S. funding acquisition; L. P.-K., S. H., A. C. R., and A. S. validation; L. P.-K., S. H., and A. C. R. investigation; L. P.-K. and S. H. visualization; L. P.-K., S. H., A. C. R., A. S., and A. R. S.-G. methodology; L. P.-K., S. H., and A. R. S.-G. writing-original draft; L. P.-K., S. D. S., and A. S. project administration; L. P.-K. and S. H. writing-review and editing.

## Footnotes

3 The abbreviations used are:

IWGAV: International Working Group for Antibody Validation
MIAPAR: Minimum Information About a Protein Affinity Reagent
BisTris: 2-[bis(2-hydroxyethyl)amino]-2-(hydroxymethyl)propane-1,3-diol
GAPDH: glyceraldehyde-3-phosphate dehydrogenase
HRP: horseradish peroxidase
PTM: post-translational modification
CGE: capillary gel electrophoresis
FBS: fetal bovine serum.
